# Structural Characterization of Mannoglucan from *Dendrobium nobile* Lindl and the Neuritogenesis-Induced Effect of Its Acetylated Derivative on PC-12 Cells

**DOI:** 10.3390/polym9090399

**Published:** 2017-08-28

**Authors:** Can Jin, Zhenyun Du, Liyan Lin, Lishuang Zhou, Saijuan Li, Qin Liu, Kan Ding

**Affiliations:** 1 School of Pharmacy, Zunyi Medical University, 201 Dalian Road, Zunyi 563003, China; Can530037655@163.com (C.J.); linliyanglc@foxmail.com (L.L.); 17602129269@163.com (L.Z.); 17602121570@163.com (S.L.); 2 Glycochemistry and Glycobiology Lab, Shangshai Institute of Materia Medica, Key Laboratory of Receptor Research, Chinese Academy of Sciences, 555 Zu Chong Zhi Road, Shanghai 201203, China; duzhenyunchina@163.com (Z.D.); liuqin@simm.ac.cn (Q.L.); 3 University of Chinese Academy of Sciences, No.19A Yuquan Road, Beijing 100049, China

**Keywords:** mannoglucan, polysaccharide, *Dendrobium nobile* Lindl, neuritogenesis, neurodegeneration disease

## Abstract

A water-soluble polysaccharide (JCS1) was isolated from the stems of *Dendrobium nobile* Lindl. JCS1 was structurally characterized using a combination of chemical and spectral analysis, including methylation analysis, partial acid hydrolysis, Fourier-transform infrared (FTIR) spectroscopy, gas chromatography (GC), GC-mass spectrometry (MS), and nuclear magnetic resonance (NMR) spectroscopy. The molecular weight was estimated to be 2.3 × 10^4^ Da using high-performance gel permeation chromatography (HPGPC). The sugar composition analysis indicated it was composed of glucose, mannose, xylose, and arabinose in a 40.2:2.3:1.7:1.0 molar ratio. The structure analysis showed that JCS1 was a mannoglucan with a backbone consisting of (1→4)-linked β-Man*p* and (1→4)-linked α-Glc*p* with branches at C-6 of (1→4)-linked α-Glc*p* residues. The branches were composed of T-α-Glc*p*, 1,4-α-Xyl*p*, and T-α-Ara*f*. In vitro bioactivity tests revealed that the acetylated derivative of JCS1, YJCS1, induced neuritogenesis of PC-12 cells. These results demonstrate that YJCS1 might be a promising bioactive polysaccharide for development as a drug candidate for the possible prevention and treatment of neurodegeneration diseases.

## 1. Introduction

*Dendrobium nobile* Lindl belongs to the orchid species and is widely distributed in Asia, including Thailand, Laos, Vietnam, and China [1,2]. It has been used as a medicinal and edible plant for thousands of years in China [2]. Its dried stems, the main medicinal part, have been studied for its “reinforcement” of body fluids, blood nourishment, promotion of saliva secretion, fever reduction, treatment of chronic gastritis, depression of cholesterol levels, and immunostimulation [2,3]. Recently, polysaccharides isolated from *D. nobile* have been demonstrated to possess immunostimulatory, antitumor, antioxidant, and hypoglycemic activities [3,4,5,6]. Attempts have been made to apply appropriate methods to modify the native polysaccharides to optimize their biological activities [7,8,9,10,11,12,13]. However, it is not clear whether acetylated modification of the native polysaccharide could induce neuritogenesis. In this paper, we report our determination of the structure of native polysaccharide obtained from *D. nobile* and the neuritogenesis-promoting effect of its acetylated derivative on PC-12 cells.

## 2. Materials and Methods

### 2.1. Materials

The dried stems of *D. nobile* were purchased from *Dendrobium nobile* Industrial Development Co., Ltd., (Guizhou, China). The purification medium Q Sepharose Fast Flow was purchased from GE Healthcare Life Science (Pittsburgh, PA, USA). The dialysis tubes with a molecular weight cutting off (MWCO) of 3500 Da were from Shanghai Green Bird Co (Shanghai, China). The different pullulans with known molecular weight (P-5, P-10, P-20, P-50, P-100, P-200, P-400, and P-800) were from Shodex Co., (Tokyo, Japan). Monosaccharide standards (mannose, rhamnose, glucuronic acid, galacturonic acid, glucose, galactose, xylose, arabinose, and fucose) were from Fluka (Buchs, Switzerland). Deuteroxide (D_2_O, 99.9% D) was from CIL (Andover, MA, USA). 3-(4,5-Dimethylthiazol-2-yl)-2,5-diphenyl tetrazolium bromide (MTT) was from Sigma-Aldrich (Saint Louis, MO, USA). PC-12 cells were from American Type Culture Collection (ATCC, Manassas, VA, USA). All reagents were of analytical grade unless otherwise noted.

### 2.2. Preparation of JCS1 and Its Acetylated Derivative

#### 2.2.1. Preparation of JCS1

The herbals of *D. nobile* (2 kg) were cut into 2 cm piece of stem and defatted by immersion in 95% ethanol (40 L) for twice, each for five days, at 25 °C. The ethanol extract were stirred once every 1 h. Five days later, the herbal residues were immersed into 40 L of ethanol again to further defatted with stirring as aforementioned. After ethanol was removed, the ethanol-insoluble residues were air dried at 25 °C. Then the residues were extracted with boiling water (40 L) seven times, 4 h each time. After filtration, the extracts were concentrated to the volume of 4 L by heating at 100 °C followed by dialysis with filter bag (cutoff is 5000 Da) against running tap water for two days. After the dialyzed extract solution was centrifuged (8000 rpm) for 5 min, the supernatant was concentrated and precipitated with four volumes of 95% ethanol with vigorous stirring. After standing the mixture overnight, the mixture was centrifuged (8000 rpm) for 5 min, the precipitate was washed successively with absolute ethanol and acetone for three times, and then dried at 40 °C in an oven to obtain the crude polysaccharide, designated as JCS. JCS (10 g) was dissolved in 200 mL distilled water, and centrifuged (8000 rpm) for 5 min. The supernatant was fractionated using a Q Sepharose Fast Flow column (0.98 L) and eluted with 1.2 L of 0.1 M sodium chloride (NaCl) solution to obtain the target polysaccharide, JCS1.

#### 2.2.2. Preparation of Acetylated Derivative

The JCS1 was acetylated using a previously described method [14]. Briefly, JCS1 (100 mg, dried under vacuum conditions overnight) was dissolved in 8 mL dimethyl sulfoxide (DMSO), stirred with a magnetic stirrer, and reacted for 24 h at room temperature to obtain a homogeneous solution. Then, 0.6 mL pyridine and 0.5 mL acetic anhydride was added sequentially to the reaction solution, the reaction was run for 2 h at 4 °C, and then it was terminated by adding distilled water, followed by dialyzation and lyophilization to obtain the acetylated derivative, YJCS1.

The acetyl group and degree of substitution (DS) of YJCS1 were determined using a previously reported method [14]. The DS values were calculated as follows:

DS = 1.62 *M*/(43 − 0.42 *M*)

where *M* = acetyl group (%, expressed as a percentage of the detected JCS1).

### 2.3. Homogeneity and Molecular Weight

The homogeneity and molecular weight were determined using a high-performance gel permeation chromatography (HPGPC) method using an Agilent 1260 Series HPLC system (Santa Clara, CA, USA) with a tandem KS-804 and KS-802 (ID 8 mm and length 300 mm, Shodex Co., Tokyo, Japan). The column temperature was at 40.0 ± 0.1 °C. The mobile phase was 0.2 M NaCl run at a flow rate of 0.8 mL/min. All samples were prepared as 0.2% (*w*/*v*) solutions in the eluent, centrifuged, and then the supernatant was analyzed using a 20 μL injection volume in each run [15].

### 2.4. Monosaccharide Composition Analysis

The monosaccharide composition was analyzed according to a previous method [15]. Briefly, samples (2 mg) were dissolved in 2 mL water (H_2_O) and hydrolyzed with the same volume of 4 M trifluoroacetic acid (TFA) in a sealed test tube at 110 °C for 4 h. The solution was repeatedly evaporated with methanol to completely remove the TFA under reduced pressure. The hydrolysate was dissolved in 2 mL H_2_O and reduced with 50 mg sodium borohydride (NaBH_4_) for 3 h at room temperature while shaking the sealed test tube occasionally. The reduction reaction was neutralized with 25% acetic acid and evaporated with methanol to obtain a dry powder, which was further dried in an oven at 100 °C for 30 min. Then, the dried residue was acetylated with 3 mL acetic anhydride (Ac_2_O) for 1.5 h at 100 °C. After evaporating to dryness with toluene, the residue was extracted with chloroform (CHCl_3_), washed three times with distilled H_2_O (1:1, *v*/*v*), and the resulting alditol acetates were analyzed using gas chromatography (GC). The GC analysis was performed using a Shimadzu GC-14B instrument (Kyoto, Japan) with a 3% OV-225-packed glass column (3.2 mm × 200 cm). The detector was flame ionization detector (FID, Kyoto, Japan). The temperatures were 250 and 240 °C for injection and detection, respectively. The flow rate of the nitrogen carrier gas was 25 mL/min, and the column temperature was maintained at 210 °C.

### 2.5. Methylation Analysis

Using a previously reported method [16], the vacuum-dried polysaccharide (10 mg) was methylated three to four times until the polysaccharide hydroxyl absorption in the infrared (IR) spectrum (Nujol) disappeared. Then, the methylated polysaccharide was hydrolyzed with 2 M TFA at 110 °C for 4 h, reduced with NaBH_4_, and finally acetylated to obtain the partially methylated alditol acetates, which were analyzed using GC-mass spectrometry (MS, HP-5 capillary column, 0.25 mm × 30 m, Santa Clara, CA, USA). The carrier gas was nitrogen, and the column temperature was gradually increased at 2 °C/min from 140 to 250 °C.

### 2.6. Fourier Transform Infrared (FTIR) and Nuclear Magnetic Resonance (NMR) Analysis

To determine the polysaccharide FT-IR spectra, the samples were first prepared in two forms. For example, the native and per-methylated polysaccharides were processed as potassium bromide (KBr) pellets and Nujol films, respectively. Furthermore, the FT-IR measurement (PerkinElmer 591B, Waltham, MA, USA) was performed in the frequency range of 4000–500 cm^−1^.

The ^1^H NMR, (^1^H)-^1^H correlation spectroscopy (COSY) and ^13^C NMR (heteronuclear single-quantum correlation [HSQC] and heteronuclear multiple-bond correlation [HMBC]) spectra were recorded at 25 °C using a Bruker AVANCE III NMR spectrometer (Karlsruhe, Germany) with acetone as the internal standard, conducted at 500 and 125 MHz, respectively. For the deuterium-exchange, the polysaccharides (30 mg) were dissolved in 0.5 mL D_2_O (99.9% D). Furthermore, 31.50 and 4.85 ppm for the ^13^C NMR (acetone) and ^1^H NMR (HDO), respectively, were used as the references to calibrate the chemical shifts.

### 2.7. Partial Acid Hydrolysis

The JCS1 (100 mg) was dissolved in 0.05 M TFA (10 mL), hydrolyzed at 100 °C for 1 h, the resulting solution was evaporated to dryness, and then dialyzed for 48 h. The collected retentate (60 mg) was further hydrolyzed with 0.1 M TFA (6 mL) at 100 °C for 1 h. Homogeneity, molecular weight, and monosaccharide composition, as well as methylation and NMR analyses of the hydrolysates (JCS1 0.1N), were performed.

### 2.8. Bioactivity Test of Polysaccharide in PC-12 Cells

The PC-12 cells (ATCC) were seeded into 12-well plates containing 50 μg/mL polylysine for 24 h, and then they were cultured in Dulbecco’s modified Eagle’s medium (DMEM) containing 1.0% horse serum and 0.5% fetal bovine serum (both serum are inactivated at 56 °C for 30 min before using) for 24 h. Subsequently, varying concentrations of polysaccharide JCS1 and YJCS1 were added to the cell culture medium and incubated for 72 h while 25 ng/mL nerve growth factor (NGF, Invitrogen, Boston, MA, USA) was used as a positive control. The cell morphology was observed using an inverted fluorescent microscope (Olympus IX73, Tokyo, Japan), and the images were acquired at a magnification of 400×. The experiments were performed in triplicate using duplicate wells.

## 3. Results

### 3.1. Isolation, Purification, and Composition Analysis

The crude polysaccharide JCS was obtained in 1.3% yield by boiling-water extraction from the dried stems of *D. nobile*, and further fractionated by anion-exchange chromatography on a Q Sepharose Fast Flow column to give 0.1 M NaCl eluent JCS1 (yield 18.9%). JCS1 was demonstrated to be a homogeneous polysaccharide by the presence of a symmetrical peak on HPGPC. The average molecular weight of JCS1 was estimated to be 2.3 × 10^4^ Da. The results showed that JCS1 contained 6.6% protein using the Lowry method [17], and was shown to be free of uronic acid using the *m*-hydroxyl diphenyl method [18]. The sugar composition of JCS1 was determined using GC analysis. The results showed that JCS1 contained glucose, mannose, xylose, and arabinose in a molar ratio of 40.2:2.0:1.3:1.0.

### 3.2. IR and Specific Rotation Analysis

**Figure 1:**
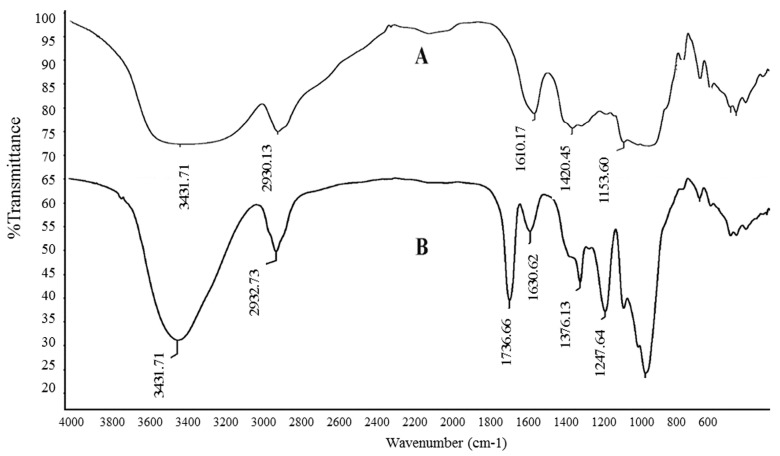
(**A**) Fourier transform infrared (FTIR) spectra of JCS1 and (**B**) acetylated derivative YJCS1.

The previously reported method used for the analysis [14] showed that JCS1 was acetylated and named YJCS1. The IR spectra of JCS1 and its acetylated derivative YJCS1 are shown in Figure 1. Typical polysaccharide signals were observed in their IR spectra. Compared with JCS1, the hydroxyl stretching band at 3431.7 cm^−1^ of YJCS1 was obviously smaller. The new absorption signals that appeared at 1247.6 and 1736.9 cm^−1^, assigned as the C=O stretching vibration, indicating that the acetylated modification of JCS1 was successful [19]. The specific optical rotation of JCS1 was estimated to be 115.3° (*c* 1.0, H_2_O).

### 3.3. Linkage Type Analysis

The analysis was performed according to a previous method [16]. The polysaccharide JCS1 (10 mg) was methylated four times after it was vacuum-dried. The JCS1 was completely methylated, hydrolyzed, reduced, and *O*-acetylated as partially methylated alditol acetates (PMAA), which were analyzed using GC-MS and the results are summarized in Table 1. The results showed that this polysaccharide had six main glycosyl residues, which were 2,3,4,6-Me_4_-Glc*p*, 2,3,6-Me_3_-Glc*p*, 2,3-Me_2_-Glc*p*, 2,3,6-Me_3_-Man*p*, 2,3-Me_2_-Xyl*p* and 2,3,5-Me_3_-Ara*f* in the molar ratio of 2.6:34.2:3.2:2.2:1.6:1.0. The results suggested that JCS1 might have a backbone, which at least consisted of (1→4)-linked-Glc*p* with branches at C-6 of (1→4)-linked-Glc*p*.

### 3.4. Partial Acid Hydrolysis and Structure Characterization of Degraded Polysaccharide

To characterize the JCS1, it was partially hydrolyzed with 0.05 M TFA and dialyzed to obtain the retentate, which was further hydrolyzed with 0.1 M TFA and dialyzed against water to provide the final retentate, named JCS10.1N. HPGPC analysis showed that JCS10.1N was homogeneous with a molecular weight of 15.5 kDa. The results of the monosaccharide composition analysis showed that JCS10.1N was composed of glucose, mannose, and xylose in a molar ratio of 40.1:2.2:1.0. It was methylated and analyzed using GC-MS, and the results are shown in Table 1. The results showed that the residues linkage types were 1,4-linked Glc*p* (17.8%), 1,4,6-linked Glc*p* (3.0%), 1,4-linked Man*p* (2.3%), and 1,4-linked Xyl*p* (0.6%), and T-linked α-Glc*p* (2.6%). Compared with the methylation analysis of the native JCS1 (Table 1), the T-linked α-Ara*f* vanished, which suggests that the Ara residues were sensitive to this mild acid. This indicates that Ara was probably located on the outer branches and that JCS1 has a (1→4)-linked Man*p* and (1→4)-linked α-Glc*p* backbone with branches at the C-6 of (1→4)-linked α-Glc*p* residues.

### 3.5. NMR Results

**Figure 2:**
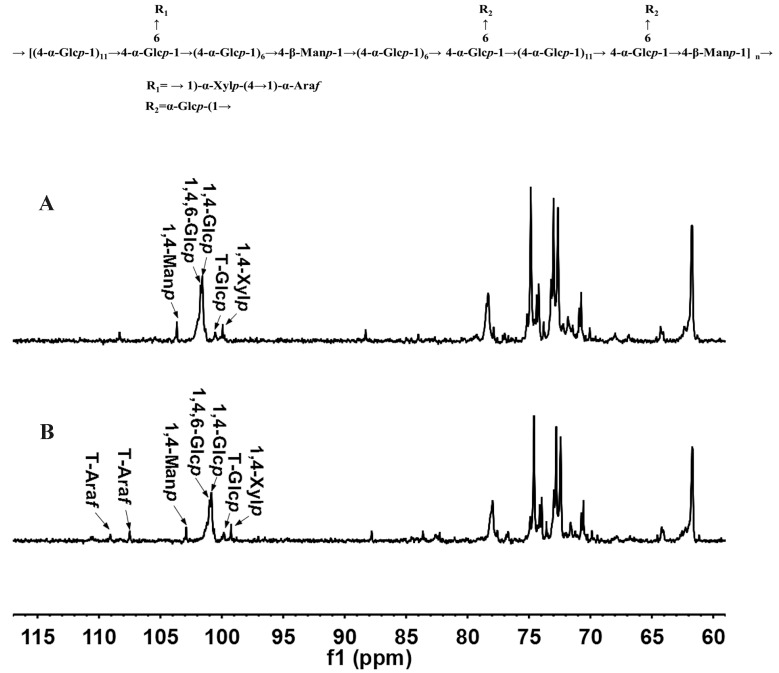
^13^C nuclear magnetic resonance (NMR) spectra of JCS1 polysaccharide and its degraded polysaccharide JCS10.1N. ^13^C NMR spectrum of (**A**) JCS10.1N and (**B**) JCS1.

**Figure 3:**
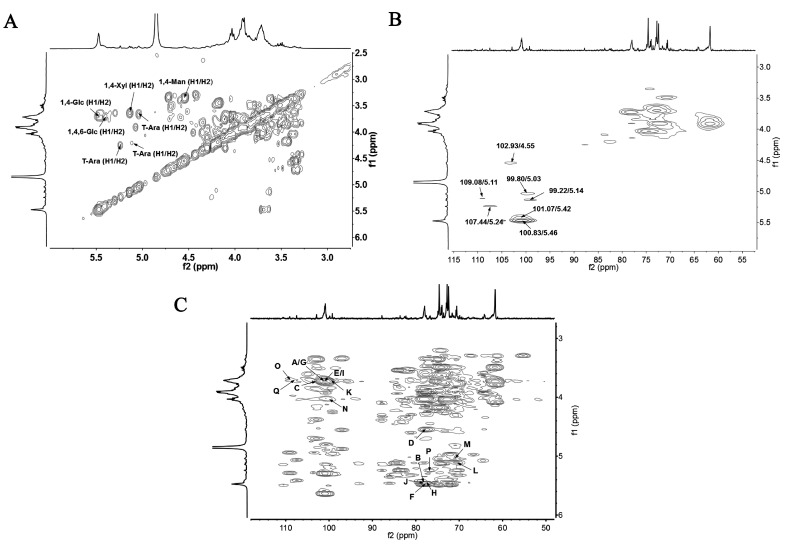
Two-dimensional spectra of JCS1. (**A**) Proton (^1^H)-^1^H correlation spectroscopy (COSY); (**B**) heteronuclear single-quantum correlation (HSQC); and (**C**) heteronuclear nuclear multi-bond correlation HMBC spectra.

The ^13^C NMR spectra of JCS10.1N and JCS1 are shown in Figure 2A,B, respectively. In the ^13^C NMR spectrum of JCS1, the anomeric signals of Glc, Man Xyl, and Ara were assigned and combined with the ^13^C NMR spectrum of degraded fractions of JCS10.1N, according to monosaccharide composition, methylation results [1,20,21,22,23,24,25,26,27]. The anomeric resonances at 100.83 and 101.07 ppm were assigned as the C1 of 1,4- and 1,4,6-linked α-Glc*p*, respectively. The anomeric resonances at 99.80, 99.22, and 102.93 ppm were assigned as the C1 of T-Glc*p*, 1,4-Xyl*p*, and 1,4-Man*p*, respectively. While the anomeric resonances at 109.08 and 107.44 ppm were assigned as the C1 of T-Ara*f* at different chemical locations. In the ^1^H NMR spectrum, the signals at 5.46 and 5.42 ppm were assigned to the anomeric H-1 of 1,4- and 1,4,6-linked α-Glc*p*, respectively according to their correlation with the anomeric carbons at 100.83 and 101.07 ppm, respectively in the HSQC (Figure 3B). Similarly, the signals at 4.55, 5.03, and 5.14 ppm were ascribed to the H-1 of 1,4-linked β-Man*p*, terminal α-Glc*p*, and 1,4-linked α-Xyl*p*, respectively (Figure 3A). The resonances at 5.11 and 5.24 ppm were attributed to the anomeric hydrogen of terminal α-Ara*f* at different chemical locations. The other resonances were also assigned, and the chemical shifts corresponding to H2 to H5 or H6 of all the residues are listed in Table 2.

In the HMBC spectrum (Figure 3C), the cross peak A (δ101.07/δ3.72) represented the correlation between C-1 of 1,4,6-linked α-Glc*p* and H-4 of the 1,4-linked α-Glc*p*. The cross peak B (δ78.02/δ5.42) represented the correlation between the C-4 of 1,4-linked α-Glc*p* and H-1 of 1,4,6-linked α-Glc*p*. The cross peak C (δ102.93/δ3.72) showed the correlation between the C-1 of 1,4-linked β-Man*p* and H-4 of 1,4-linked α-Glc*p*. The cross peak D (δ78.02/δ4.55) showed the correlation between the C-4 of 1,4-linked α-Glc*p* and H-1 of 1,4-linked β-Man*p*. The cross peak E (δ100.83/δ3.72) indicated the correlation of the C-1 of 1,4-linked α-Glc*p* and H-4 of 1,4-linked β-Man*p*. The cross peak F (δ5.46/δ77.58) represented the correlation between the H-1 of 1,4-linked α-Glc*p* and C-4 of 1,4-linked β-Man*p*. The cross peak G (δ3.72/δ101.07) indicated the correlation of the H-4 of 1,4-linked β-Man*p* and C-1 of 1,4,6-linked α-Glc*p*. The cross peak H (δ77.58/δ5.42) showed the correlation between C-4 of 1,4-linked β-Man*p* and H-1 of 1,4,6-linked α-Glc*p*. The cross peak I (δ100.83/δ3.72) represented the correlation between the C-1 of 1,4-linked α-Glc*p* and H-4 of 1,4,6-linked α-Glc*p*. The cross peak J (δ5.46/δ78.02) represented the correlation between H-1 of 1,4-linked α-Glc*p* and C-4 of 1,4,6-linked α-Glc*p*. The cross peak K (δ99.22/δ3.72) represented the correlation between the C-1 of 1,4-linked α-Xyl*p* and H-6 of 1,4,6-linked α-Glc*p*. The cross peak L (δ5.14/δ70.40) showed the correlation between the H-1 of 1,4-linked α-Xyl*p* and C-6 of 1,4,6-linked α-Glc*p*. The cross peak M (δ5.03/δ70.40) showed the correlation between the H-1 of T-α-Glc*p* and C-6 of 1,4,6-linked α-Glc*p*. The cross peak N (δ99.80/δ4.02) indicated the correlation of the C-1 of T-α-Glc*p* and H-6 of 1,4,6-linked α-Glc*p*. The cross peak O (δ107.44/δ3.72) showed the correlation between C-1 of T-α-Ara*f* and H-4 of 1,4-linked α-Xyl*p*. The cross peak P (δ5.24/δ76.70) indicated the correlation of H-1 of T-α-Ara*f* and C-4 of 1,4-linked α-Xyl*p*. The cross peak Q (δ109.08/δ3.72) showed the correlation between C-1 of T-α-Ara*f* at different chemical locations and H-4 of 1,4-linked α-Xyl*p*. According to the above results, the backbone of JCS1 might consist of repeated 1,4-linked β-Man*p* and 1,4-linked α-Glc*p* units with branches at the C-6 of 1,4-linked α-Glc*p* substituted by 1,4-linked α-Xyl*p* and T-α-Ara*f* linked at C-4 of 1,4-linked α-Xyl*p*. The other branches might be linked by T-α-Glc*p* at C-6 of 1,4-linked α-Glc*p*. Based on the monosaccharide analysis, methylation, partial hydrolysis, and NMR analysis, a possible repeating unit for JCS1 was proposed as follows:

### 3.6. YJCS1 Induces Neurite Extension of PC-12 Cells

Considerable research has been carried out to investigate the antioxidant and immunomodulatory activity of acetyl derivatives of polysaccharides [24,28,29]. The acetylation (DS) of YJCS1 was 0.025 while the average molecular weight was 18.8 kDa.

**Figure 4:**
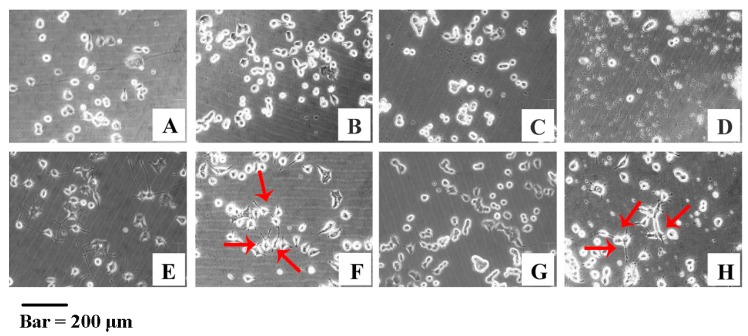
The PC-12 cells were treated with (**A**) normal medium or with different concentrations of polysaccharide JCS1; (**B**) 5.56; (**C**) 33.33; and (**D**) 55.56 μM for 120 h; (**E**) PC-12 cells treated with nerve growth factor (NGF) as a positive control; (**F**–**H**) PC-12 cells incubated with YJCS1 at three different concentrations; (**F**) 5.56; (**G**) 33.33; and (**H**) 55.56 μM. Represented positive neuritogenesis induced by YJCS1 was indicated by red arrows.

Interestingly, we found that JCS1 did not extend the neurites of the PC-12 cells; however, the induction of the PC-12 cells was observed to be in a dose-dependent manner following treatment with the acetyl derivative of JCS1. The induced neuritogenesis was significant at low and high concentrations (Figure 4F,H). The results suggested that this acetylated polysaccharide had the potential to be developed as a drug candidate to prevent or even treat neurodegeneration diseases.

## 4. Discussion

In recent decades, differences have been discovered in the molecular weight, category, and molecular ratios of monosaccharide linkage types of polysaccharides extracted from *Dendrobium* plant species. However, there might be some common structural characteristics among *Dendrobium* plant polysaccharides. For example, 1,3- and 1,4-linked mannose residues are usually in the β configuration [1,23,30,31,32] while 1,2- and 1,6-linked mannose residues are in the α configuration [23,32]. Most galactose residues are terminal linkages, which are in the α configuration [1,4,23]. Few 1,3- and 1,6-linked galactose residues are the backbone [3,4]. Glucose residues are 1,4- and 1,6-linked and are basically on the backbone [1,4,23,30,31,32]. In addition, all kinds of *Dendrobium* have neutral polysaccharides [1,3,4,23,30,31,32], but interestingly, no studies have reported that *Dendrobium* has pectic polysaccharide [2]. This is likely because most *Dendrobium* polysaccharides have been extracted from its stems, which have a lower acidic polysaccharide content than that of the fruit body and flowers. Furthermore, the monosaccharide composition of a few homogeneous polysaccharides consists of low quantities of arabinose and xylose. Both of these two residues are on the branches [2,27].

Based on the structure-activity relationship analysis, the diverse structural characteristics of *Dendrobium* polysaccharides have resulted in various bioactivities. The first detected bioactivity of polysaccharides from *Dendrobium officinale* was an immunomodulatory activity [33]. Other kinds of *Dendrobium* polysaccharides were also discovered to have immunostimulatory properties [1,2,3,23,24,25,26,27,28,29,30,31,32]. In addition, some researchers have focused on the antioxidant and antitumor activities.

DCPP1a-1, isolated from the suspension-cultured protocorms of *Dendrobium* significantly inhibited hydroxyl radicals and superoxide anion radicals [34]. In another study [26], mice injected intraperitoneally with *Dendrobium denneanum* polysaccharide for 20 days showed lower serum malondialdehyde (MDA) and higher serum superoxide dismutase (SOD) than the control mice did. Some polysaccharides inhibit tumor cell growth in vitro [35] and in vivo [33,36]. The researchers considered the antitumor properties to be associated with the excellent immune-enhancing and antioxidant properties of the polysaccharide [33]. Moreover, considerable research has been focused on studying other bioactivities of *dendrobium* polysaccharides. For instance, the DCLP polysaccharide isolated from *Dendrobium chrysotoxum* had antidiabetic effects in alloxan-induced hyperglycemic mice [37]. In addition, the DHP-W2 polysaccharide from *Dendrobium huoshanense* might impede anti-glycation activity [27].

Currently, some acetylated polysaccharides from *Dendrobium* have been reported [1,2,23,30,31]. These polysaccharides were extracted from *D. nobile*, *D. officinale*, and *D. huoshanense*. The substitution position of their acetyl groups differed, and all the acetylated *Dendrobium* polysaccharides had bioactivities. It was observed that the acetyl groups were just substituted at the O-2 or O-3 of glucose and mannose [1,23,30,31]. Similarly, considerable research has shown that acemannan, an acetylated polymannose from *Aloe vera*, promoted tissue repair [38,39]. Furthermore, some investigations were carried out on the acemannan to determine the effect of the acetyl-groups on its physical and biological properties [40,41]. Previous studies have demonstrated the important role of acetyl groups on polysaccharide bioactivity [40]. The above research studies have provided two enlightening pieces of information or questions. One is that the bioactivities are different between the native active *Dendrobium* polysaccharides with acetyl groups and those without acetyl groups, which were deacetylated. Another important question is whether acetylated derivatives of novel *Dendrobium* polysaccharides, which mainly contain glucose or mannose have novel bioactivities. Therefore, in this study, the novel polysaccharide containing abundant glucose extracted from *D. nobile* was acetylated. Moreover, the acetylated polysaccharide, YJCS1, induced the neuritogenesis of PC-12 cells, which provides a reference for studying novel bioactivities of acetylated derivatives of *Dendrobium* polysaccharides.

## Figures and Tables

**Table 1:** Linkage analysis of JCS1 polysaccharide and its degraded product JCS10.1N using gas chromatography (GC-MS).

Methylated Sugars	Linkages	Molar Ratio %	
		JCS1	JCS10.1N *
2,3,6-Me_3_-Glc	1,4-Glc*p*	34.2	17.8
2,3,4,6-Me_4_-Glc	T-Glc*p*	2.6	2.6
2,3-Me_2_-Glc	1,4,6-Glc*p*	3.2	3.0
2,3,6-Me_3_-Man	1,4-Man*p*	2.2	2.3
2,3-Me_2_-Xyl	1,4-Xyl*p*	1.6	0.6
2,3,5-Me_3_-Ara	T-Ara*f*	1.0	_

* JCS10.1N was polysaccharide dialyzed retentate from JCS1 after it was partially hydrolyzed with 0.05 and 0.1 M TFA, respectively.

**Table 2:** Proton (^1^H) and ^13^C nuclear magnetic resonance (NMR) spectral assignments for JCS1 (ppm).

Residues		1	2	3	4	5	6
1,4-α-Glc*p*	H	5.46	3.71	4.02	3.72	3.73	3.90
	C	100.83	72.75	74.53	78.02	72.45	61.57
T-α-Glc*p*	H	5.03	3.68	3.83	3.49	3.79	3.90
	C	99.80	71.94	74.06	69.84	72.76	61.65
1,4,6-α-Glc*p*	H	5.42	3.69	4.00	3.72	3.99	3.72/4.02
	C	101.07	71.46	73.50	78.02	74.93	70.40
1,4-β-Man*p*	H	4.55	3.36	3.85	3.72	3.84	3.75
	C	102.93	73.95	73.61	77.58	71.51	64.09
1,4-α-Xyl*p*	H	5.14	3.68	3.71	3.72	3.72	-
	C	99.22	73.61	73.02	76.70	61.25	-
T-α-Ara*f*	H	5.11	4.20	4.03	4.12	3.46	-
	C	109.08	82.57	76.66	83.56	64.07	-
T-α-Araf	H	5.24	4.26	nd	nd	nd	-
	C	107.44	nd	nd	nd	nd	-

nd: not detectable.
